# Nanozyme-based sensing of dopamine using cobalt-doped hydroxyapatite nanocomposite from waste bones

**DOI:** 10.3389/fbioe.2024.1364700

**Published:** 2024-04-17

**Authors:** Umar Nishan, Nighat Jabeen, Amir Badshah, Nawshad Muhammad, Mohibullah Shah, Irfan Ullah, Saifullah Afridi, Jibran Iqbal, Muhammad Asad, Riaz Ullah, Essam A. Ali, Sarfraz Ahmed, Suvash Chandra Ojha

**Affiliations:** ^1^ Department of Chemistry, Kohat University of Science and Technology, Kohat, Pakistan; ^2^ Department of Dental Materials, Institute of Basic Medical Sciences Khyber Medical University, Peshawar, Pakistan; ^3^ Department of Biochemistry, Bahauddin Zakariya University, Multan, Pakistan; ^4^ Department of Neurology, Khyber Teaching Hospital Peshawar, Peshawar, Pakistan; ^5^ College of Interdisciplinary Studies, Zayed University, Abu Dhabi, United Arab Emirates; ^6^ Department of Pharmacognosy, College of Pharmacy, King Saud University Riyadh Saudi Arabia, Riyadh, Saudi Arabia; ^7^ Department of Pharmaceutical Chemistry, College of Pharmacy, King Saud University Riyadh Saudi Arabia, Riyadh, Saudi Arabia; ^8^ Wellman Center for Photomedicine, Harvard Medical School, Massachusetts General Hospital, Boston, MA, United States; ^9^ Department of Infectious Diseases, The Affiliated Hospital of Southwest Medical University, Luzhou, China

**Keywords:** cobalt-doped hydroxyapatite, hydrogen peroxide, peroxidase mimic, dopamine, colorimetric sensor

## Abstract

Dopamine is one of the most important neurotransmitters and plays a crucial role in various neurological, renal, and cardiovascular systems. However, the abnormal levels of dopamine mainly point to Parkinson’s, Alzheimer’s, cardiovascular diseases, etc. Hydroxyapatite (HAp), owing to its catalytic nature, nanoporous structure, easy synthesis, and biocompatibility, is a promising matrix material. These characteristics make HAp a material of choice for doping metals such as cobalt. The synthesized cobalt-doped hydroxyapatite (Co-HAp) was used as a colorimetric sensing platform for dopamine. The successful synthesis of the platform was confirmed by characterization with FTIR, SEM, EDX, XRD, TGA, etc. The platform demonstrated intrinsic peroxidase-like activity in the presence of H_2_O_2_, resulting in the oxidation of 3,3′,5,5′-tetramethylbenzidine (TMB). The proposed sensor detected dopamine in a linear range of 0.9–35 μM, a limit of detection of 0.51 µM, limit of quantification of 1.7 µM, and an R^2^ of 0.993. The optimization of the proposed sensor was done with different parameters, such as the amount of mimic enzyme, H_2_O_2_, pH, TMB concentration, and time. The proposed sensor showed the best response at 5 mg of the mimic enzyme, pH 5, 12 mM TMB, and 8 mM H_2_O_2_, with a short response time of only 2 min. The fabricated platform was successfully applied to detect dopamine in physiological solutions.

## 1 Introduction

Dopamine is an important neurotransmitter in the human body, and it is implicated in various debilitating diseases. It plays a central role in the renal, neurological, cardiovascular, hormonal, and metabolic systems (Dhanasekaran et al., 2018). Dopamine plays an important role in the brain’s ability to receive signals, and low levels of it can cause a variety of neurological diseases, including epilepsy, schizophrenia, and Parkinson’s disease (Howes et al., 2017). Its normal level ranges from 1.3 to 2.6 μM. Beyond the given limits, it can point to heart diseases, Parkinson’s disease, cardiovascular problems (Nishan et al., 2020), abnormal thyroid hormone levels, neuromuscular problems, and schizophrenia (Kienast and Heinz, 2006). The correlation between dopamine and several devastating and fatal illnesses underscores the need to sense it accurately, selectively, and cost-effectively.

In recent years, various sophisticated methods have been reported for the sensing of dopamine, such as fluorescence (Wang et al., 2015), electrochemistry (Xu et al., 2016), chemiluminescence (Xu et al., 2011), high-performance liquid chromatography (Carrera et al., 2007), electrochemiluminescence (Wang et al., 2017), etc. Despite their merits, the majority of these techniques require complex sample pretreatment. These techniques are not only expensive to acquire and sustain but also laborious and time-consuming. The need for highly skilled operators is another major limitation of the aforementioned techniques. Background interference and low reproducibility have also been reported for the mentioned methods. These shortcomings associated with the reported techniques put a question mark on their viability as techniques of choice for dopamine sensing and biosensing. Therefore, much work needs to be done to provide straightforward, quick, and effective techniques for the high-sensitivity detection of dopamine. Colorimetric assays offer a viable alternative in comparison to the above-stated techniques. They have attracted considerable attention owing to their naked-eye observation capability, easy operation, and lower cost (Nishan et al., 2021). Moreover, the naked eye observation can further be confirmed through a UV-Vis spectrophotometer for accurate quantification (Shi et al., 2016). Natural enzyme-based sensing platforms have been used for the detection of various biomarkers, but they are expensive, have a low shelf life, are difficult to handle, are temperature-sensitive, and lose their activity under harsh conditions (Fang et al., 2006).

The emergence of mimetic enzymes (nanozymes) is a new viable alternative to overcome the limitations of natural enzymes (Zhu et al., 2017). Nanozymes are artificial enzymes based on nanomaterials that mimic the role of natural enzymes. Their superior catalytic properties and ability to endure harsh conditions have impressed researchers (Jiang et al., 2019). They are also used in bioassays (Zhu et al., 2017), as industrial catalysts (Maurya et al., 2015), in food processing (Oueslati et al., 2018), agriculture (Anjum et al., 2016), and environmental monitoring (Lu et al., 2013). Mimic enzymes offer the advantages of lower cost, high stability, a large surface area, and easy operation (Han et al., 2015). Transition metal-based enzyme mimics are the most effective class of artificial enzymes owing to their high conductivity and different oxidation states. It has been reported that Co−Fe_3_O_4_/graphene (Zhu et al., 2018), Co_3_O_4_@NiO (Hosseini et al., 2017), CoFe_2_O_4_/CoS (Yang et al., 2018), and CoS nanospheres (Luong et al., 1988) function as good peroxidase mimetic catalysts with favorable catalytic activity and high stability. Cobalt nanoparticles have been extensively used as nanozymes for sensing and catalytic activities due to their high stability and low cost. However, the problem of agglomeration limits their use as reliable players in the fabrication of sensing platforms for bioanalysis (Nana et al., 2021). Researchers have used various strategies to overcome the problem of agglomeration in metal nanoparticles.

For this purpose, in most cases, metal nanoparticles are mixed with other functional elements to provide a synergistic effect. Different matrix materials have been used to achieve the deagglomeration of the metal nanoparticles. Hydroxyapatite (HAp) has fantastic osteointegrative and osseoconductive properties. It is a nanoporous substance that is biocompatible, catalytic (owing to the presence of basic and acidic moieties), bioactive, degradable, and ubiquitous (Roopalakshmi et al., 2017). These qualities make it a popular candidate for its use as a matrix material for the fabrication of metal-based mimic enzymes (Irfan and Irfan, 2020). It can easily be synthesized from waste materials such as bovine bone (Barua et al., 2019), fish bone (Muhammad et al., 2016), coral (Pountos and Giannoudis, 2016), egg shells, etc (Gergely et al., 2010). HAp is a promising material for the fabrication of such platforms due to its exceptional ability to withstand a wide range of cationic and anionic substituents (Fihri et al., 2017). Doping with different kinds of metal ions improves the physical, chemical, and biological properties of HAp (Ratha et al., 2021).

In this work, we have used cobalt-doped HAp nanocomposite as a peroxidase mimic for the colorimetric sensing and biosensing of dopamine for the first time. Co-doped HAp, as a mimic enzyme, can catalyze the oxidation of TMB with the assistance of H_2_O_2_. The colorless solution changes to a blue-green product in the presence of H_2_O_2_, acting as an oxidizing agent. This transformation can be observed with the naked eye and confirmed with a UV-Vis spectrophotometer. As dopamine was added to the reaction mixture, it reduced the oxidized TMB to TMBred, with the consequent disappearance of the blue-green color to colorless. The fabricated sensing platform was successfully applied in a physiological solution for the sensing of dopamine.

## 2 Experimental

### 2.1 Chemicals and materials

CoCl_2_.6H_2_O, dopaime, NaOH, HCl, 3,3′,5,5′-Tetramethylbenzidine (TMB), DMSO, H_2_O_2_, 35%, and NaH_2_PO_4_ were purchased from Sigma-Aldrich. Double-distilled water was used for the preparation of solutions. Bio-World-provided phosphate-buffered saline (pH 7.4) was used in the reactions as a medium.

### 2.2 Instrumentation

Using Fourier transform infrared spectroscopy on the Cary 630 FTIR spectrometer (Agilent Technologies, Danbury, Connecticut, USA), the distinctive peaks of HAp and Co-HAp were characterized. The materials’ FTIR spectra were obtained within the given range of 4,000–400 cm^−1^. The parameters set for FTIR experiments were 256 scans per sample and 4 cm^−1^ resolution. Scanning electron microscopy linked to energy dispersive X-ray spectroscopy (SEM-EDX) using a TESCAN VEGA (LMU) SEM with an INCAx-act (Oxford Instruments) EDX attachment working at 20 kV was used to analyze the morphology of the produced materials. The produced materials’ phase was investigated using X-ray diffraction (Shimadzu, LabX XRD-6100 with Cu-Kα radiation) with a scan range of 10°–80°. The voltage for acceleration was set at 30 kV, while the current was set at 20 mA. Cu Kα radiations were utilized with a monochromatic wavelength of (λ = 1.5405 Å). Using JCPDS, file No. 04–0783, the phase of the produced HAp and Co-HAp nanocomposite was identified. Under nitrogen gas flow, thermal gravimetric analysis (TGA) was performed at temperatures between 50°C and 800°C at a heating rate of 10°C/min. Using a UV-Vis spectrophotometer (Shimadzu, UV, 1,800, Japan), absorption spectra were captured.

### 2.3 Preparation of HAp from waste bones

The waste bones of goats were used to prepare HAp. The bones were boiled for two to 3 hours to remove the remains of organic components. To ensure further cleaning, the bones were treated with an acetone solution (70% v/v solution in water) for 3 hours. Water was used to rinse the bones several times. The treated bones were dried for 24 h at 100°C in an oven. The dried bones were pulverized with the help of a pestle and mortar. To obtain the desired HAp, calcination was carried out at 800°C for 3 hours in a furnace (Khawar et al., 2019). The work received ethical approval from the concerned forum of Kohat University of Science and Technology, KUST, Kohat via No. KUST/Ethical Committee/1023.

### 2.4 Preparation of Co-HAp nanocomposite

**SCHEME 1:**
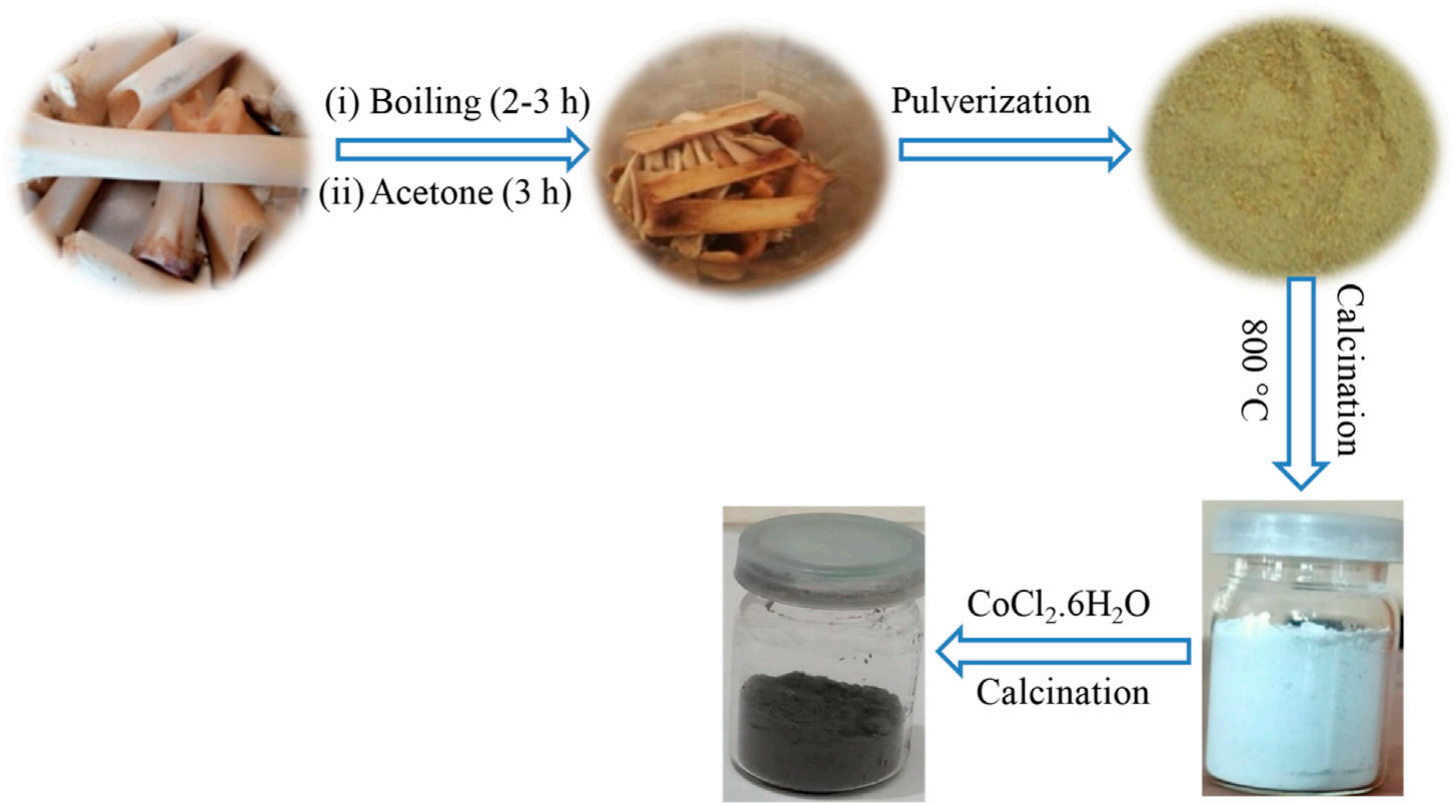
Schematic representation of the synthesis of HAp and Co-HAp.

Cobalt (II) chloride hexahydrate (CoCl_2_.6H_2_O) was mixed with the synthesized HAp powder for doping at a weight ratio of 1:9, respectively. The ingredients were mixed with a pestle and mortar to form a homogenous mixture. The mixture was calcined at 800°C for 3 hours in a furnace. The overall process for the preparation HAp and Co-HAp is shown in the Scheme 1.

### 2.5 Dopamine sensing through Co-HAp nanocomposite

The synthesized Co-HAp nanocomposite (5 mg) was suspended in a 500 μL PBS solution. At the same time, 100 μL of TMB solution (12 mM in DMSO) and 100 μL of H_2_O_2_ (8 mM) were added to the solution mixture. The catalytic ability was measured by recording the changes in absorbance over time and acquiring the absorption spectra. TMB was used as a substrate for the colorimetric detection of dopamine, and the peroxidase-like activity was measured. The expected colorimetric change was confirmed using both the naked eye and UV-Vis spectra.

In order to validate the catalytic role of Co-HAp in the reaction system, the sensing of dopamine was performed under different conditions. These include TMB + Co-HAp, TMB + H_2_O_2_, and TMB + H_2_O_2_+Co-HAp.

### 2.6 Sensing of dopamine in physiological solution

The physiological solution was utilized to detect dopamine. In this solution, three different concentrations of dopamine were spiked. In the prepared samples, colorimetric detection of dopamine was performed and confirmed by a UV-Vis spectrophotometer (Zheng et al., 2011).

## 3 Results and discussion

### 3.1 Characterization of HAp and Co-HAp nanocomposite

#### 3.1.1 FTIR analysis of the prepared HAp and Co-HAp nanocomposite

**FIGURE 1:**
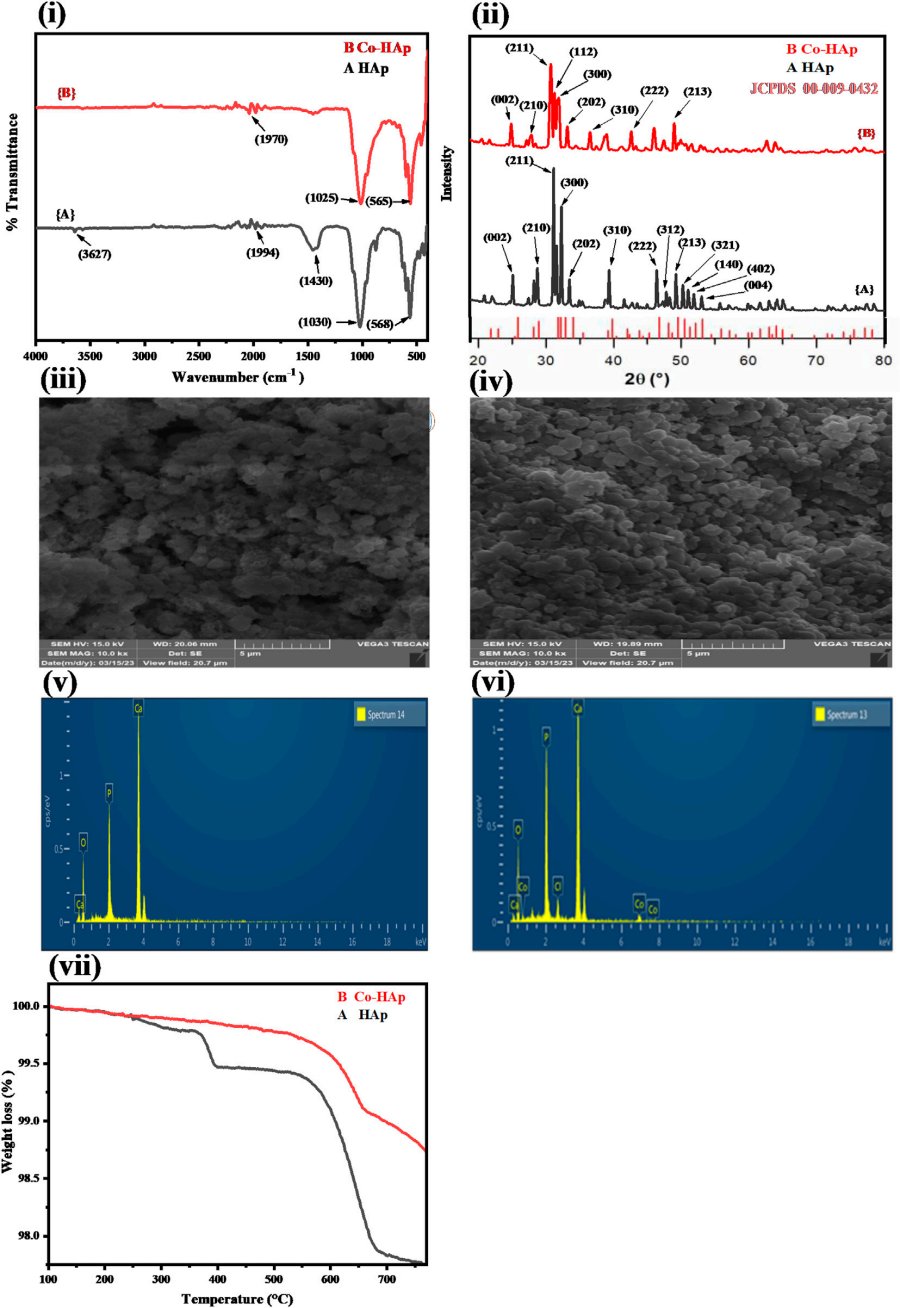
**(i)** FTIR spectra for HAp **(A)** and Co-HAp nanocomposite **(B)**, indicating the presence of characteristic peaks in the synthesized materials. **(ii)** XRD pattern of the HAp **(A)** and Co-HAp nanocomposite **(B)**, showing broadening of the peaks in doped HAp. SEM image of the HAp **(iii)** and Co-HAp nanocomposite **(iv)**, indicating the spherical shape and nanoporous morphology of the synthesized platform. EDX analysis of the HAp **(v)** and Co-HAp nanocomposite **(vi)** shows the presence of Co along with other elements. **(vii)** TGA thermogram of the prepared HAp **(A)** and Co-HAp nanocomposite **(B)**, demonstrating its thermal stability in the temperature range of 100–800°C.

Figure 1i demonstrate the FTIR spectra of the pure HAp and Co-HAp nanocomposite. The FTIR spectrum of the synthesized HAp nanoparticles indicated peaks at 568, 1030, 1430, and 3,627 cm^−1^. The peak around 568 cm^−1^ represents the bending mode of phosphate functionality, while the band at 1030 cm^−1^ indicates the stretching mode of the phosphate moiety. The peak in the range of 1400–1600 cm^−1^ shows the presence of a carbonate group in the HAp. The peak at 3,627 cm^−1^ represents the hydroxyl group that comes from moisture. Co-HAp nanocomposite indicated peaks at 565 cm^−1^ and 1025 cm^−1^. These peaks represent the phosphate functionality in the synthesized nanocomposite (Sahana et al., 2013). There is no more peak of carbonate functionality due to the removal of carbonyl functionality in the form of carbon dioxide. However, there is no significant difference between the pure and Co-doped HAp (Yazdani et al., 2019).

#### 3.1.2 XRD analysis of the prepared HAp and Co-HAp

Figure 1ii displays the XRD pattern of the synthesized HAp and Co-HAp. Using the JCPDS database Card-No. (09–0432), (Palierse et al., 2022), the distinct diffraction peaks were compared based on the anatase HAp and Co-HAp phases. According to the standard data sheet the XRD pattern of the doped samples 1 (B) was found to be comparable to that of crystalline HAp (Ca_10_(PO_4_)_6_(OH)_2_). With the exception of a minor widening of the peaks at 32°, 33°, and 34° 2θ, the XRD profiles of the doped HAp did not change significantly from those of pure HAp and did not show any new peaks belonging to cobalt. The probable anionic replacement of OH ions in the HAp by chloride ions of cobalt chloride, in addition to the replacement of calcium by cobalt, might be the cause of this drop in crystallinity. Crystal lattice distortion, crystallite size, and percentage crystallinity are the factors that influence peak broadening (Khaliq et al., 2023). Using the Scherrer equation for the anatse phase of HAp and Co-HAp and the most intense peak, the average particle size was determined (Nachit et al., 2016). The average crystal sizes of the anatase phases of HAp and Co-HAp were estimated to be about 38 and 32 nm, respectively.

#### 3.1.3 SEM analysis of the prepared HAp and Co-HAp

Figure 1iii represents the SEM image of HAp and Co-HAp nanocomposite (iv). The HAp and Co-HAp nanocomposites show a spherical morphology. A decrease in particle size was observed with the doping of cobalt into the HAp structure, which increases its surface area. Both pure and doped HAp show nanoporous structures, which can be helpful in providing the necessary surface area for catalytic reactions.

#### 3.1.4 EDX analysis of prepared HAp and Co-HAp

Figure 1v, vi; Table 1 show the elemental composition of HAp and Co-HAp. The EDX spectrum of pure HAp shows the presence of calcium, oxygen, and phosphorus in the weight percentage of 37.78, 49.97, and 12.25, respectively, as shown in Table 1. Whereas Co-HAp, along with the already present elements calcium (32.59%), oxygen (45.32%), and phosphorus (16.0%), shows the presence of chlorine and cobalt in the weight percentages of 2.19 and 3.90, respectively. This confirms the successful doping of Co on the surface of the synthesized HAp.

**TABLE 1:** EDX analysis by weight of the synthesized HAp and Co-HAp nanocomposite.

Element	HAp		Co-Hap	
	Weight %	Atomic %	Weight %	Atomic %
P	12.25	8.86	16.00	12.04
Ca	37.78	21.13	32.59	18.59
O	49.97	70.01	45.32	66.02
Cl	---	---	2.19	1.44
Co	---	---	3.90	1.91
Total	100.00	100.00	100.00	100.00

#### 3.1.5 TGA analysis of prepared HAp and Co-HAp

In order to ascertain the performance of the fabricated platform in harsh thermal conditions, it is essential to determine its thermal stability. Figure 1vii shows the thermal gravimetric analysis study of the synthesized HAp and Co-HAp nanocomposite. The TGA of pure HAp from 200 to 400°C is 0.601% weight loss, and from 500 to 800°C, the weight loss is 1.612%. The overall weight loss in pure HAp is 2.213%. The TGA of Co-HAp weight loss is 1.172%; it is from 500 to 800°C. There is no considerable weight loss in Co-HAp because HAp is already thermally stable even at higher temperatures.

### 3.2 Proof of catalytic activity of the mimic enzyme

**FIGURE 2:**
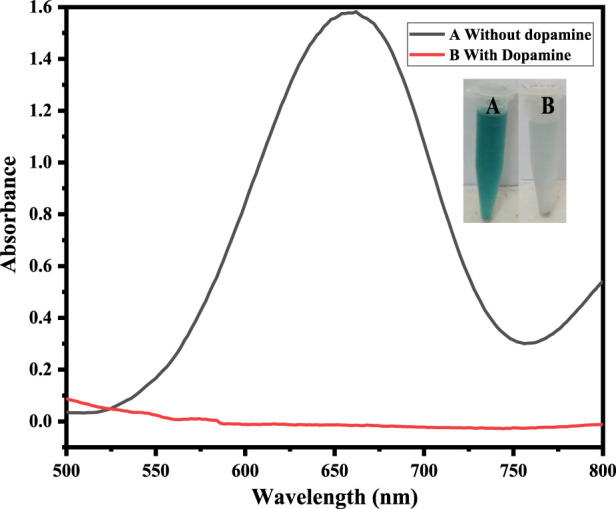
Shows that at (A) 5 mg Co-HAp nanocomposite, 100 µL TMB (12 mM), 500 µL PBS, 100 µL H_2_O_2_ (9 mM), and (B) shows that the addition of 100 µL dopamine (35 µM) results in the reduction of the oxidized TMB.

The Co-HAp nanocomposite demonstrated improved peroxidase-like catalytic activity. Using the peroxidase substrate TMB and H_2_O_2_, the peroxidase-like catalytic activity of the Co-HAp nanocomposite was investigated. The shift in absorbance of the oxidized TMB (TMB_oxi_) at 652 nm was monitored to observe the progress of the reaction. It is clear from Figure 2 that there is no oxidation when the TMB and Co-HAp are present only in the system. There is a very little oxidation of TMB when H_2_O_2_ is present. However, the combination of H_2_O_2_ and Co-HAp, significant TMB oxidation of takes place (Ivanova et al., 2019). It is clearly evident from this that H_2_O_2_ and Co-HAp are both necessary for the oxidation of TMB. This suggests that the catalyst accelerates the oxidation of TMB in the presence of H_2_O_2_ by exhibiting peroxidase-like activity.

### 3.3 Colorimetric sensing of dopamine

**FIGURE 3:**
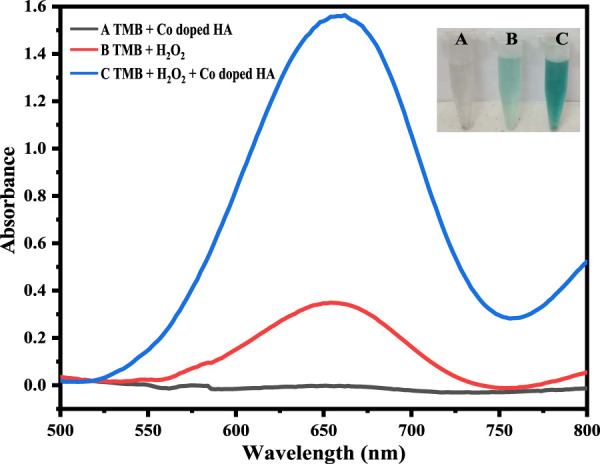
The Figure demonstrates different catalytic systems, that is, in the presence of Co-HAp only (A), H_2_O_2_ only (B), both H_2_O_2_ and Co-HAp (C), 5 mg of Co-HAp nanocomposite, 100 µL TMB sol (12 mM), 500 µL PBS (pH 5), 100 µL H_2_O_2_ (9 mM), and a temperature of 25°C).

The fabricated sensing platform, i. e., Co-HAp nanocomposite (5 mg), was used for dopamine colorimetric sensing, with TMB serving as a chromogenic substrate. In a typical experiment, 100 µL of hydrogen peroxide (9 mM) was combined with 100 µL of TMB (12 mM) in 500 µL of PBS solution. The color changed from transparent to blue-green, with a visible colorimetric change (A). After the addition of 100 µL of dopamine (35 µM), the blue-green color changed to transparent (B) due to the reduction of oxidized TMB, as confirmed by the UV-Vis spectrophotometer. Inset Figure 3 shows both the colorimetric change and the UV-Vis spectra.

### 3.4 Sensing mechanism of dopamine via Co-HAp nanocomposite

**SCHEME 2:**
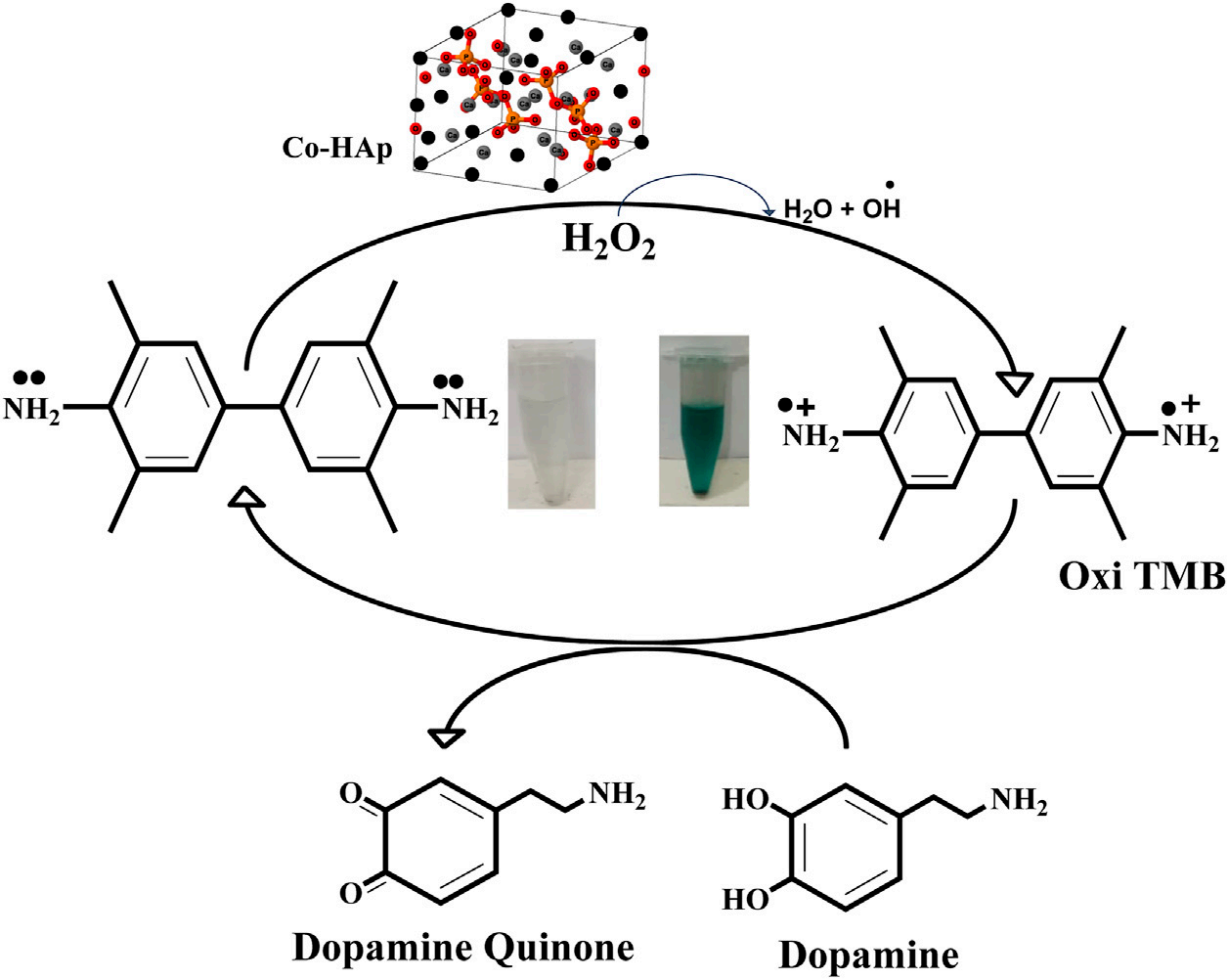
A schematic representation of the proposed mechanism shows the oxidation of the chromogenic substrate and its subsequent reduction as a result of dopamine addition to the reaction mixture.

The mimic enzyme (Co-HAp nanocomposite) can work as an excellent platform for the sensing of dopamine. In this process, the oxidizing potential of hydrogen peroxide assisted the mimic enzyme in the oxidation of TMB. A visible colorimetric change to a blue-green color occurs, indicating the oxidation of TMB. Upon the addition of dopamine, the reaction complex changes from blue-green to transparent. This colorimetric change was confirmed through a UV-visible spectrophotometer. The catalytic action was based on a remarkable color change from colorless. The oxidation of TMB to a blue-green color is mediated by a hydroxyl radical that is generated as a result of hydrogen peroxide breakdown assisted by the mimic enzyme. The generated hydroxyl free radical removes the electron from the amino group of the TMB and oxidizes it. Here, the OH free radical acts as an oxidizing agent, and the resulting oxidized TMB gives a blue-green color to the reaction mixture. In reverse, the addition of dopamine to the reaction mixture reduces the oxidized TMB to its original colorless form and itself oxidizes to dopamine quinone (Zheng et al., 2021). A detailed mechanism is shown in Scheme 2.

### 3.5 Optimization of various parameters

#### 3.5.1 Effect of Co-HAp nanocomposite amount

**FIGURE 4:**
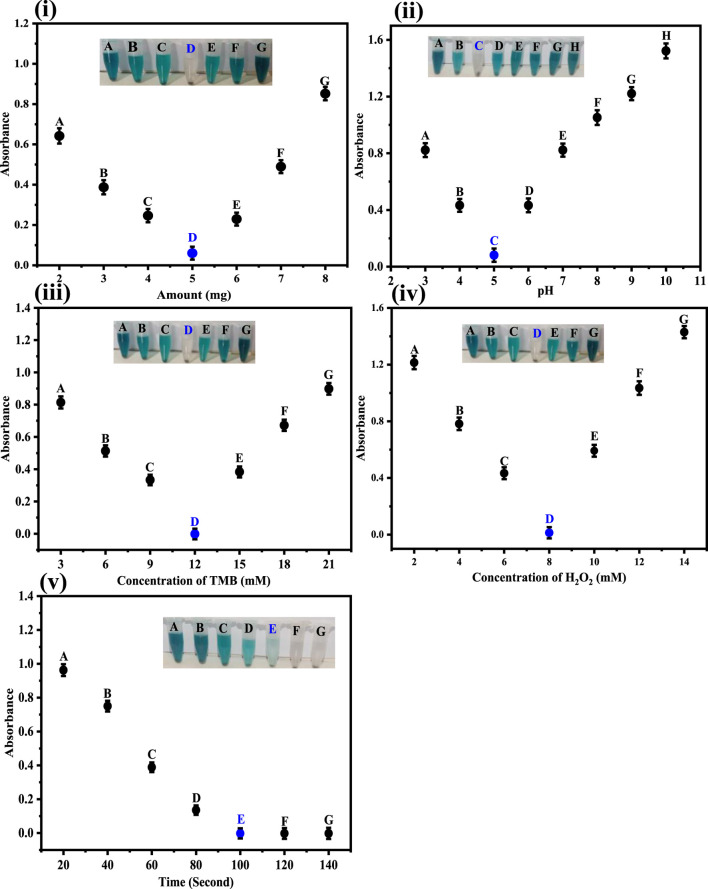
**(i)** Optimization of the amount of Co-HAp in mg. The best response was observed at 5 mg of Co-HAp PBS 500 μL, TMB 100 µL (12 mM), H_2_O_2_ 150 µL (8 mM), and dopamine 100 µL (35 µM). **(ii)** Optimization of pH. The best response was observed at pH 5. Conditions: [Co-doped HAp loading] = 5 mg, [dopamine conc.] = 3.68 × 10^−7^ M. **(iii)** Optimization of the TMB concentration in mM. The best response was observed under 12 mM TMB conditions: [H_2_O_2_ conc] = 8 mM (150 µL), [dopamine conc.] = 35 µM (100 µL). **(iv)** Optimization of the H_2_O_2_ conc. in mM. The best response was observed at 8 mM. Conditions: [TMB conc.] = 12 mM (100 µL), [dopamine conc.] = 35 µM (100 µL). **(v)** Optimization of time with respect to absorption obtained from UV-Vis spectra. Condition: [Co-doped HAp] = 5 mg; [TMB conc.] = 12 mM; [H_2_O_2_ conc.] = 8 mM; [dopamine conc.] = 35 µM.

In order to assess the effect of Co-HAp, different amounts in the range of 2–8 mg of the proposed platform were evaluated. As the amount of Co-HAp increased, the color of the solution mixture started to fade. The solution color completely vanished at 5 mg of the Co-HAp. With the increase in the concentration of Co-Hap, the color of the reaction mixture starts to intensify to blue-green. For these experiments, 100 µL of TMB solution (12 mM), 500 µL of phosphate-buffer saline, 100 µL of H_2_O_2_, and 100 µL of a 35 µM dopamine solution were taken. Under the prevailing conditions, the reaction took only 2 min. Ivanova et al. reported 7 mg of mimic enzyme as optimal in their work (Ivanova et al., 2019). Figure 4i illustrates the correlation between different Co-HAp nanocomposite concentrations, at given amounts of TMB, H_2_O_2_, and dopamine.

#### 3.5.2 Impact of pH on the fabricated sensor

The fabricated sensing platform was tested in the pH range 3–10, as shown in Figure 4ii. As the pH increases from three to five, the color of the chromogenic substrate gradually vanishes until it completely disappears at pH 5. As the pH increased further, the color of the reaction mixture appeared again and intensified untill it reached pH 10. In these experiments, 5 mg doped Co-HAp nanocomposite, 100 μL TMB solution (12 mM), incubation time 2 min, 100 μL H_2_O_2_, and 100 μL dopamine solution (35 µM) were used. As a result, a pH of five was determined to be the ideal pH for the suggested probe. pH four was proposed as the optimal pH for the work reported in an earlier published work (Ivanova et al., 2019).

#### 3.5.3 Effect of TMB concentration

The optimization results for TMB concentration for the sensing of dopamine are shown in Figure 4iii. The TMB concentration was varied in the range of 3–21 mM in the optimization experiments. It is clear from the inset Figure that at 12 mM of TMB concentration we get the best response. A TMB concentration, lower or higher than the mentioned amount does not produce any desirable results. In these experiments, 100 µL of TMB (3–21 mM), 100 µL of H_2_O_2_ (9 mM), and 5 mg of doped Co-HAp nanocomposite were used. Wu et al. reported 0.5 mM TMB as optimal in their work (Wu et al., 2018). In the subsequent experiments, 12 mM of TMB concentration was used.

#### 3.5.4 Effect of H_2_O_2_ concentration

In order to obtain the optimal H_2_O_2_ concentration, the sensing experiments were performed at different concentrations of H_2_O_2,_ ranging from 2 to 14 mM, as shown in Figure 4iv. Results indicate that the best response was achieved at 8 mM of H_2_O_2_. These experiments were performed under the following conditions: 5 mg of Co-HAp nanocomposite; 100 µL of TMB (12 mM); and 500 µL of PBS. Yang et al. reported 65 mM to be the optimal concentration of H_2_O_2_ in their work (Yang et al., 2018).

#### 3.5.5 Time optimization

Figure 4v depicts the optimization of time for the proposed sensing platform. The maximal efficiency of the Co-HAp nanocomposite was observed after 2 minutes. Under optimal conditions, the proposed sensor detects dopamine in 2 min. Further increases in time show no change in color, indicating the significance of the fabricated platform. An earlier study reported that 7 min are required for the detection of the analyte (Wang et al., 2019).

### 3.6 Analytical merits of the method

**FIGURE 5:**
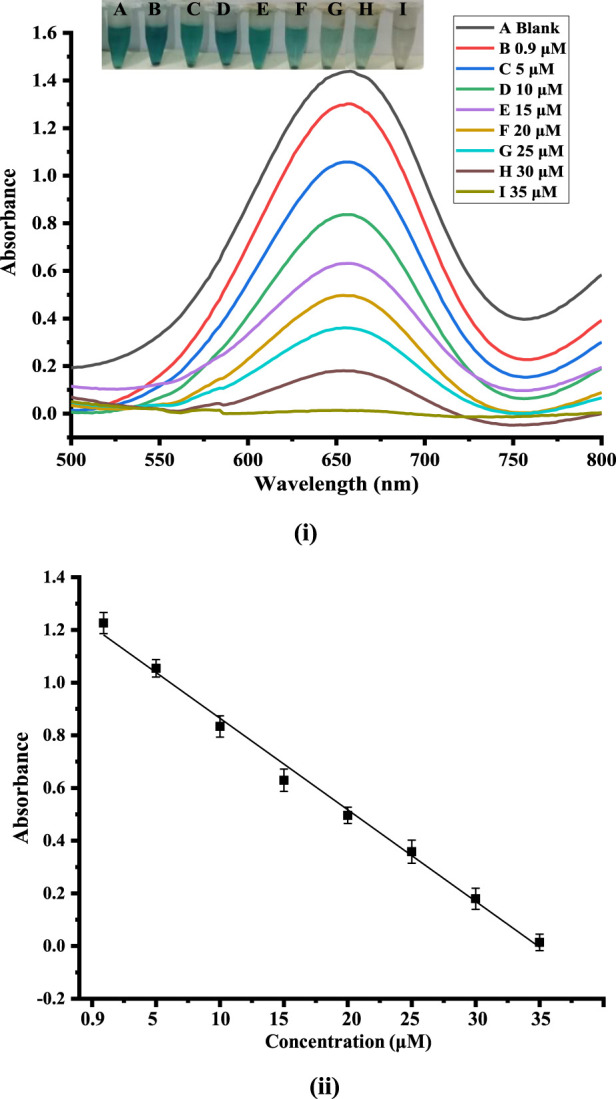
**(i)**: Showing the calibration plot of absorbance *versus* dopamine concentration. **(ii)** UV-Vis spectra and the alterations that correlate to variations in dopamine concentrations.

An easy and quick colorimetric detection method was used to detect dopamine under the ideal experimental conditions. Based on the catalytic activity of the synthesized Co-HAp nanocomposite, the sensor’s sensitivity to dopamine detection was examined. Various dopamine concentrations were used during the evaluation of the fabricated platform for its analytical merits. As seen, with the increasing concentration of dopamine, results the UV-Vis peak at 652 nm diminishes. Different concentrations of dopamine were taken in the range of 0.9–35 μM, as shown in Figures 5i, ii. In the absence of dopamine, it shows a peak at 652 nm in the UV spectrum and decreases linearly as dopamine concentration rises. Dopamine detection was performed in a linear range of 0.9–35 µM with an LOD of 0.51 µM and an LOQ of 1.7 µM with regression coefficient (R^2^) value of 0.993.

### 3.7 Comparing the suggested sensor with different methods

As indicated in Table 2, the efficacy of the suggested sensor was evaluated by contrasting the findings with those of previous investigations. The comparison shows the excellent performance of the proposed sensor in terms of lower LOD and wide linear range.

**TABLE 2:** A brief comparison of our synthesized platform with the reported sensors for the colorimetric detection of dopamine.

S.No	Analytical method	Linear range (µM)	LOD (µM)	References
1	Pt/CoFe_2_O_4_	20–80	0.42	He et al. (2020)
2	CoFe_2_O_4_/CoS	0–50	0.58	Yang et al. (2018)
3	Au NP-PET	0.5–500	0.5	Rostami et al. (2020)
4	G-ZIF-8	3–1000	1.0	Zheng et al. (2017)
5	(NiO) NPs	2–100	1.038	Roychoudhury et al. (2016)
6	TGA-CdTe QDs	3–100	1.3	Nejad and Hormozi-Nezhad (2017)
7	(CuS-BSA-Cu_3_(PO_4_)_2_)	0.05–100	0.13	Swaidan et al. (2021)
8	CuS-rGO	2–100	0.48	Dutta et al. (2015)
9	Co−Fe_3_O_4_/graphene	0.5–50	0.08	Hosseini et al. (2017)
10	Co_3_O_4_@NiO	1–1000	1.21	Zhu et al. (2018)
11	Pt/CoSn(OH)_6_	5.0 to 60	4.42	Liu et al. (2019)
12	Co-HAp	0.9–35	0.51	This work

### 3.8 Interference studies

**FIGURE 6:**
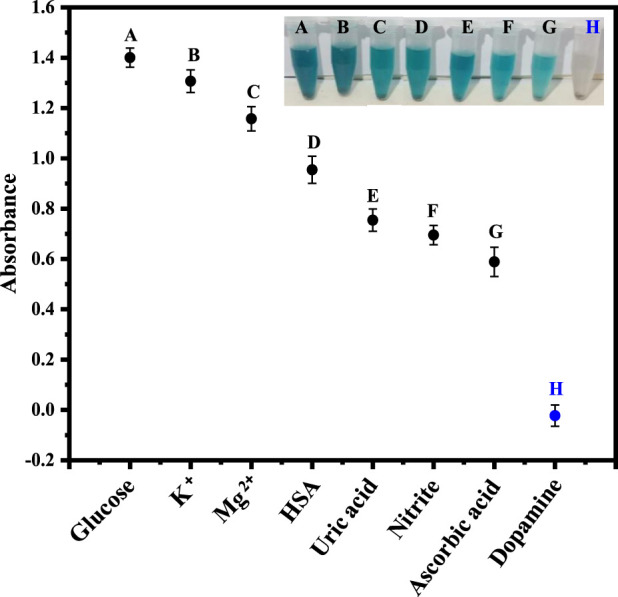
The Figure demonstrates the selectivity of the fabricated sensor for dopamine in the presence of other analytes, including glucose, K^+^, Mg^2+^, HSA, uric acid, nitrite, and ascorbic acid.

Under optimized conditions, the fabricated platform was tested for the selective sensing of dopamine. For this purpose, glucose, K^+^, Mg^2+^, human serum albumin (HSA), uric acid, nitrite and ascorbic acid, were examined as potential interfering species, as shown in Figure 6. The findings demonstrate that dopamine has a relatively low absorption value as compared to the other species.

### 3.9 Application of the fabricated sensor in physiological solution

**FIGURE 7:**
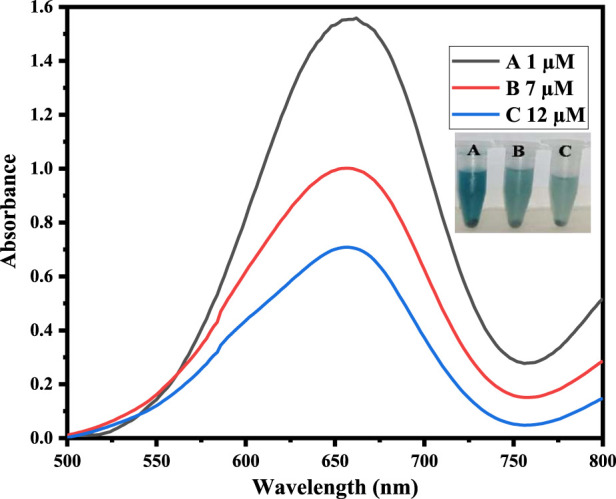
Physiological sample analysis by adding different concentrations of dopamine in physiological solutions.

In order to examine the use of the suggested platform, we applied the technique for dopamine detection in physiological solutions. For this purpose, a physiological solution was used to analyze dopamine. Physiological solutions of three different concentrations of dopamine were prepared for sensing through the fabricated platform. As the dopamine concentration increases, the intensity of the absorption peak at 652 nm decreases linearly. The colorimetric change was observed in a short time of only 2 min. As shown in Figure 7, the sensor is very efficient and exceptionally sensitive for detecting dopamine in biological samples.

## 4 Conclusion

In conclusion, we have successfully fabricated a new Co-HAp sensing platform for the colorimetric sensing of dopamine. All the characterizations confirmed the successful synthesis of HAp and Co-HAp. The synthesized mimic enzyme, with the synergistic effect of hydrogen peroxide, oxidized the TMB with a visible colorimetric change that was subsequently confirmed with a UV-Vis spectrophotometer. The addition of dopamine to the reaction mixture resulted in the reduction of the oxidized TMB to TMB_red_ and the disappearance of the blue-green color. The fabricated platform (Co-HAp) was highly sensitive, quick, and selective for the sensing of dopamine as compared to the previously reported methods. The fabricated platform was used for the sensing of dopamine in physiological solutions. The proposed sensor has the potential to be used as a laboratory tool for the diagnosis, management, and monitoring of various neurological disorders at low cost and easy operation.

## Data Availability

The original contributions presented in the study are included in the article/Supplementary material, further inquiries can be directed to the corresponding authors.
